# Clinical significance of accurate identification of lymph node status in distant metastatic gastric cancer

**DOI:** 10.18632/oncotarget.6009

**Published:** 2015-10-19

**Authors:** Rui Zhou, Zhenzhen Wu, Jingwen Zhang, Hongqiang Wang, Yuqi Su, Na Huang, Min Shi, Jianping Bin, Yulin Liao, Wangjun Liao

**Affiliations:** ^1^ Department of Oncology, Nanfang Hospital, Southern Medical University, Guangzhou 510515, China; ^2^ Department of Oncology, Zhoushan Hospital, Zhoushan 316000, China; ^3^ Department of Oncology, The First People's Hospital of Yueyang, Yueyang 414000, China; ^4^ Department of Cardiology, Nanfang Hospital, Southern Medical University, Guangzhou 510515, China

**Keywords:** lymph node status, palliative surgery, LND, distant metastatic gastric cancer, survival

## Abstract

**PURPOSE:**

The clinical consequences of accurately identifying lymph node (LN) status in distant metastatic gastric cancer (DMGC) are unclear. We aimed to determine the prognostic significance of N stage, positive LN (PLN) count, and the positive LN ratio (LNR). We also retrospectively compared survival outcomes of DMGC patients stratified by LN dissection (LND).

**RESULTS:**

LND was performed in 1593 patients. The CSS was significantly different between groups divided according to N stage, PLN, and LNR in DMGC patients who underwent LND. Lower LNR was an independent predictor of longer survival in all kinds of patients cohorts, whereas PLN was not such a predictor. PLN count correlated with LND number and LNR. No correlation existed between LNR and LND number. Undergoing LND and having a higher number of dissected LNs were associated with superior CSS.

**MATERIALS AND METHODS:**

Data from 1889 DMGC patients treated between 2004 and 2009, and documented in the Surveillance, Epidemiology, and End Results (SEER) registry, were reviewed. Pearson's correlation coefficient and the Chi-square test were used to study the relationships between LND number, PLN count, N stage, and the LNR. Cancer-specific survival (CSS) was evaluated using Kaplan-Meier analysis, with the log-rank test performed for univariate analysis (UVA) and the Cox proportional hazards model employed for multivariate analysis (MVA).

**CONCLUSION:**

LN metastatic variables play important roles in the prognostic evaluation and treatment decisions of DMGC patients. Accurate identification of LN status in DMGC patients is critical. LND performance is associated with increased survival and has clinical practicability.

## INTRODUCTION

Lymph node (LN) metastasis is one of the most common outcomes for M0 gastric cancer (GC) patients [1–4]. However, its clinical impact on distant metastatic gastric cancer (DMGC) patients remains unclear. With advances in medical technologies, surgical therapies for distant metastases are gaining attention and are linked to survival benefits [5–7]. They enable LN dissection (LND) and evaluation in DMGC patients. Therefore, it is important to determine whether accurate identification of LN metastatic status in DMGC patients is clinically worthwhile.

LN metastasis variables include N stage, [8, 9] positive LN (PLN) count, [10, 11] and LN ratio (LNR), [10, 12–15] which refers to the ratio of the PLN count to the total number of LNs dissected. These variables have all been investigated as prognostic factors in M0 GC patients, but few studies have evaluated their prognostic value in patients with DMGC. Moreover, only 3 studies based on the Surveillance, Epidemiology, and End Results (SEER) database investigated the outcomes of DMGC, [7, 16, 17] none of which systematically discussed the role of LND and LN metastatic status in the management of this disease.

The objective of the present study was to determine whether accurate identification of LN status in DMGC patients is of clinical value. We also evaluated the survival impact of LND and whether there is a minimum number of dissected LNs required to best predict overall status. To guarantee a sufficient follow-up period, our study included patients documented in the SEER database (which was administered by the National Cancer Institute) who were diagnosed between 2004 and 2009. This is the first report of its kind to be based on data extracted from the SEER database.

## RESULTS

### Patient selection and clinicopathological characteristics of the entire cohort

**Figure 1:**
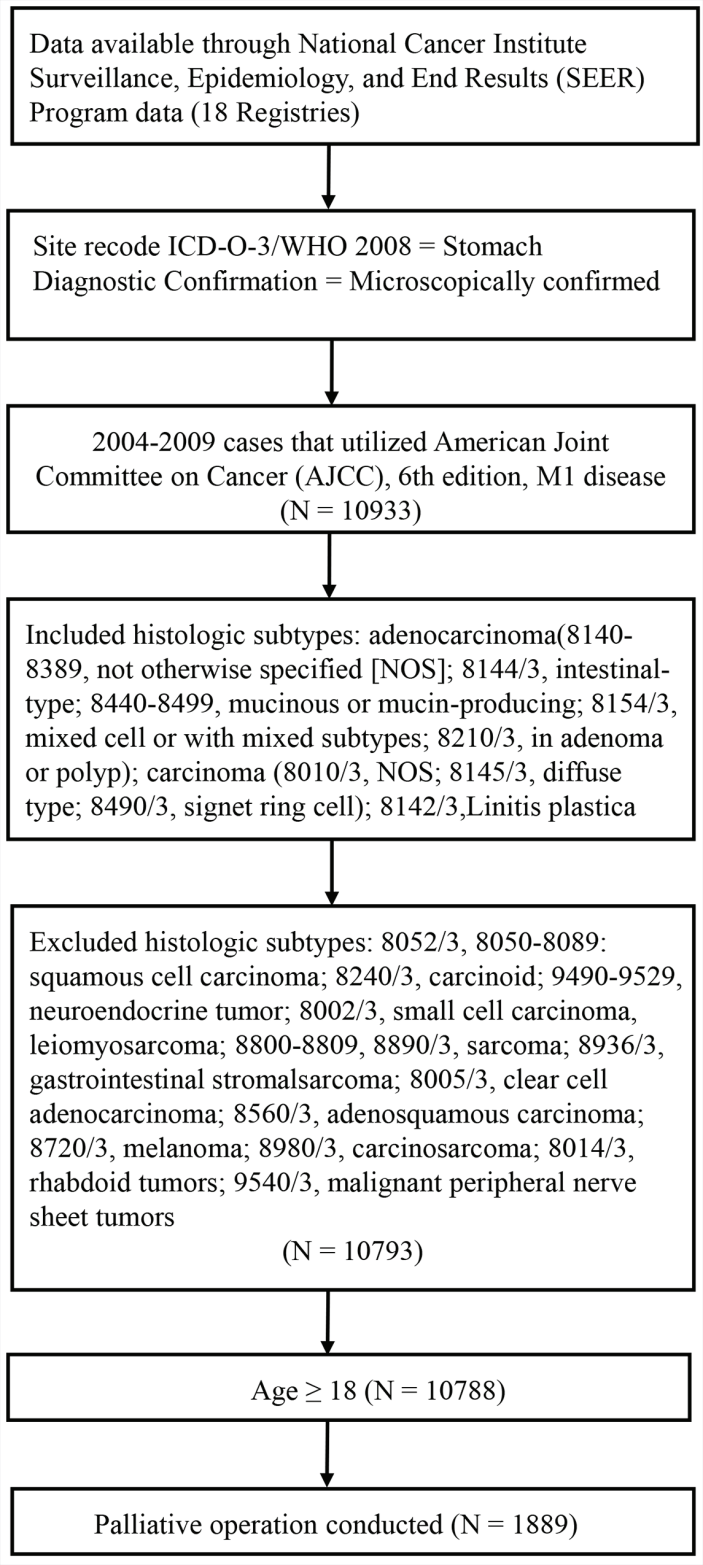
Selection of the distant metastatic gastric cancer patients included in the study

The patient selection schema is shown in Figure 1, and detailed patient characteristics are listed in Supplementary Table S1. Briefly, 1889 patients were included in this study. The median age at diagnosis was 65 years (range, 19–95 years). Median survival was 10 months and the 3-year survival rate (YSR) was 15.5%. Data from 500 (26.5%) patients were censored. LN dissection was performed in 1593 patients (84.3%). The median number of LNs examined was 13 (range, 1–90+), the median PLN count was 7 (range, 0–79), and the median LNR was 66.7%.

### Comparison of clinicopathological characteristics of the cohorts stratified by LND

Supplementary Table S2 compares the clinicopathological variables between those who underwent LND (*n* = 1593) and those who did not (*n* = 272). Compared to patients who underwent LND, those who did not undergo dissection were more likely to have lower N stages as well as a tumor histology indicating unspecified carcinoma, advanced tumor extension, and metastasis involving organs or the peritoneum. There were no significant differences regarding other variables between the subgroups.

### Survival impact of N stage, PLN, and LNR

**Figure 2:**
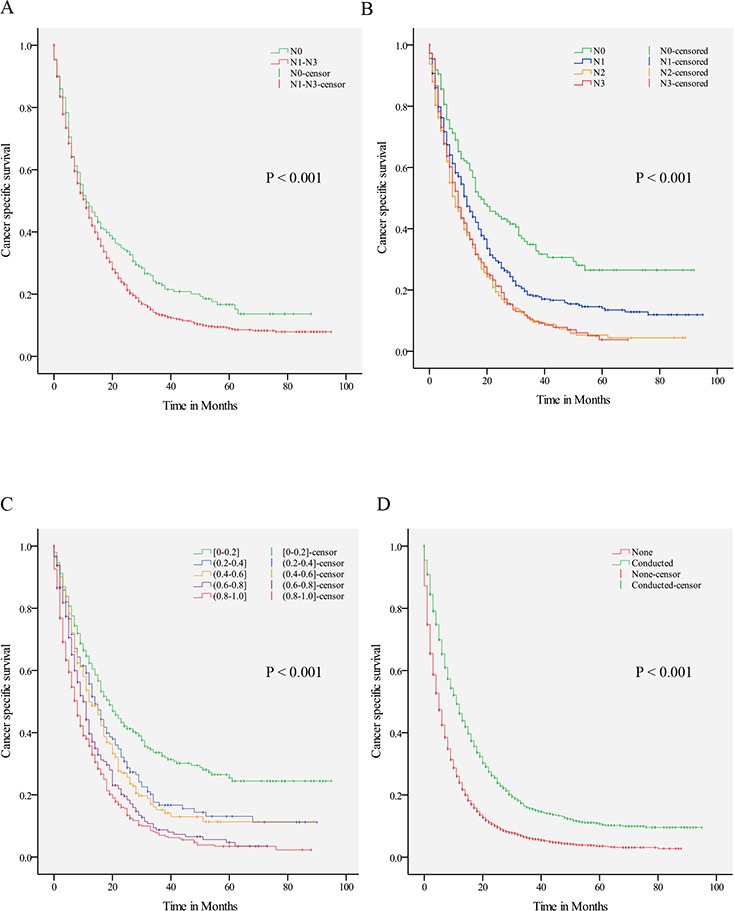
Kaplan-Meier curves of CSS by nodal metastatic status, N stage, lymph node ratio and undergoing of lymph node dissection Figure 2 shows the comparison of actuarial cancer specific survival curves in distant metastatic gastric cancer (DMGC) patients according to nodal metastatic status, N stages, lymph node ratio (LNR), and undergoing of lymph node dissection (LND). **A.** Nodal metastatic status in all DMGC patients; **B.** N stage in patients with LND; **C.** LNR in patients who underwent LND; **D.** LND in all DMGC patients.

The survival impact of N stage, PLN, and LNR are shown in Table 1, Table 2, and Figure 2. Concerning N stage (Table 1A), we found that DMGC patients with N0 stage had significantly better prognosis than non-N0 patients on univariate analysis (UVA). Such statistically significant differences were observed in all patients (Figure 2A) and in patients who underwent LND (Figure 2B) but not in those who did not (*P* = 0.206). Further analysis showed that, in patients who underwent LND, the prognosis of N0 patients was significantly more favorable than that of N1 patients, while N1 patients in turn had significantly better prognosis than N2 and N3 patients. There was no significant difference between N2 and N3 patients.

**Table 1:** Univariate analysis of the impact of metastatic lymph node variables on survival

A. Survival impact of N stages in all distant metastatic gastric cancer (DMGC) patients, DMGC patients without nodal dissection alone, and DMGC patients with nodal dissection alone.									
Cohortsof all patients	All patients			Without LND			With LND		
	MS (m)	3-YSR (%)	*P*	MS (m)	3-YSR (%)	*P*	MS (m)	3-YSR (%)	*P*
**N stage**									
N0	13.0	25.5	Ref.	9.0	13.0	Ref.	18.0	35.1	Ref.
N1–N3	10.0	14.2	0.000	7.0	5.1	0.206	11.0	14.6	0.000
N1	12.0	17.8	0.000	7.0	5.1	0.206	12.0	19.4	0.000
N2	9.0	10.6	0.036	NA	NA	NA	9.0	10.7	0.000
N3	10.0	11.2	0.011	NA	NA	NA	10.0	11.2	0.000
Abbreviations: LND, lymph node dissection; MS, median survival; m, months; YSR, year survival rate; Ref, reference; NA, not applicable.									

B. Survival impact of lymph node ratio in all DMGC patients with nodal dissection, patients with positive nodes, and patients of same N stages.															
Patients with LND	All patients			N1–N3			N1			N2			N3		
	MS (m)	3-YSR (%)	*P*	MS (m)	3-YSR (%)	*P*	MS (m)	3-YSR (%)	*P*	MS (m)	3-YSR (%)	*P*	MS (m)	3-YSR (%)	*P*
**LNR**						0.000									
[0–0.2]	19.0	34.0	Ref	19.0	32.3	Ref.	19.0	33.5	Ref.	NA	NA	NA	NA	NA	NA
(0.2–0.4]	14.0	18.6	0.002	14.0	18.8	0.014	13.0	20.0	0.015	16.0	13.5	Ref.	NA	NA	NA
(0.4–0.6]	12.0	16.4	0.000	12.0	16.4	0.002	12.0	14.6	0.006	12.0	14.0	0.503	18.0	30.8	Ref.
(0.6–0.8]	11.0	9.4	0.000	11.0	9.4	0.000	11.0	10.6	0.001	9.0	10.7	0.101	11.0	6.0	0.059
(0.8–1.0]	8.0	8.5	0.000	8.0	8.5	0.000	8.0	6.2	0.000	7.0	9.1	0.003	8.0	9.2	0.009
**LNR**						0.000									
[0–0.6]	16.0	24.7	Ref.	15.0	21.8	Ref.	15.0	24.3	Ref.	13.0	13.3	Ref.	21.0	32.7	Ref.
(0.6–1]	8.0	8.8	0.000	8.0	8.8	0.000	8.0	7.6	0.000	8.0	9.6	0.000	9.0	8.3	0.004
Abbreviations: LND, lymph node dissection; MS, median survival; m, months; YSR, year survival rate; LNR, lymph node ratio; Ref, reference; NA, not applicable.															

**Table 2:** Impact of positive lymph node count and lymph node ratio on survival in distant metastatic gastric cancer patients by multivariate analysis

Item	All patients		N1–N3		N1		N2		N3	
	HR	95% CI	HR	95% CI	HR	95% CI	HR	95% CI	HR	95% CI
Age^a^	1.006*	1.001–1.010	1.005*	1.000–1.010	NS		NS		NS	
T stage (vs. T0–T2)	NS		NS		NS		NS		NA	
Grade (vs. I–II)	1.205*	1.017–1.429	NS		NS		NA		NA	
Tumor site (vs. Body)										
Fundus	NS		NS		NA		2.733**	1.313–5.688	NA	
Tumor extent (vs. Localized)										
Regional	NS		NS		NS		1.329*	1.003–1.761	NS	
Further extent	1.605**	1.215–2.120	1.568**	1.173–2.096	NS		1.695*	1.062–2.705	2.022*	1.156–3.534
Mets at diag (vs. DNs)										
OPI	1.266**	1.061–1.511	NS		NS		NS		NA	
OPI and DNs	1.326*	1.014–1.735	NS		NS		NS		NA	
Radi & Surg (vs. Surgery alone)	0.731**	0.611–0.874	0.663**	0.547–0.804	0.613**	0.460–0.817	0.658**	0.482–0.898	NA	
PLN^a^	0.997	0.989–1.004	0.997	0.989–1.004	1.033	0.973–1.097	1.015	0.971–1.060	1.006	0.993–1.019
LNR^a^	2.384**	1.920–2.960	2.408**	1.885–3.077	2.123**	1.533–2.941	2.241**	1.386–3.624	5.640**	2.320–14.264

Survival was analyzed in all patients with nodal dissections, patients with positive nodes and patients of same N stages.

a Continuous variable

* *P* < 0.05

** *P* < 0.01.

Abbreviations: HR, hazard ratio; CI, confidence interval; Mets, metastasis status; diag, diagnosis; OPI, organs or peritoneal involved; DNs, distant nodes; PLN, positive lymph node; LNR, lymph node ratio; NA, not applicable; NS, not significance; Radi, radiation; Surg, surgery.

The LNR (Figure 2C, Table 1B) was negatively correlated with patients' median survival and 3-YSR. For binary classified LNR, cancer-specific survival (CSS) was more favorable in those with lower LNR values in all the cohorts we analyzed. For multi-category LNR (those who underwent LND, those with LN metastasis [N1–N3], and those of N1 stage), the median survival and 3-YSR significantly decreased as the LNR increased. In patients of N2 or N3 stage, a statistically significant difference was noted although it was not uniformly distributed.

When determining the prognostic significance of PLN and LNR by multivariate analysis (MVA) (Table 2), we found that the LNR was highly predictive of worse CSS in all patient categories, whereas PLN count was not.

### Correlations between the number of LNs examined, the PLN count, N stage, and LNR

**Figure 3:**
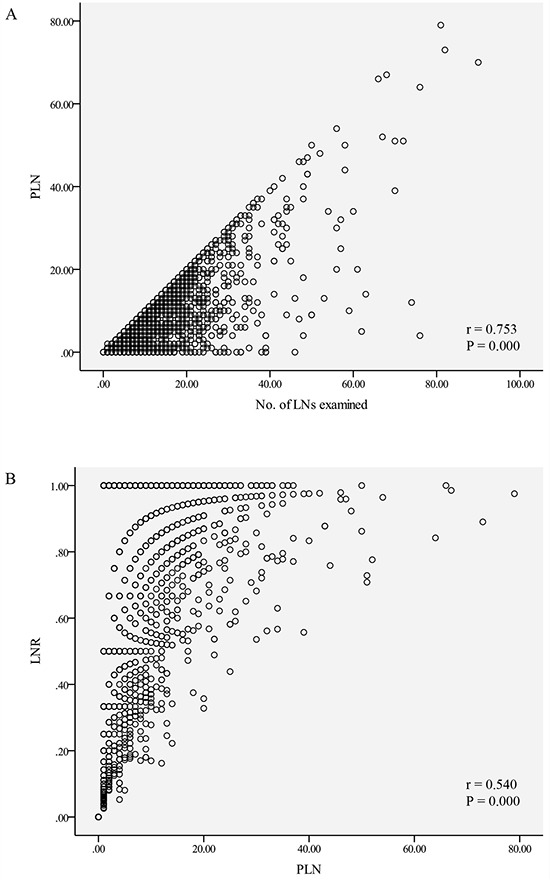

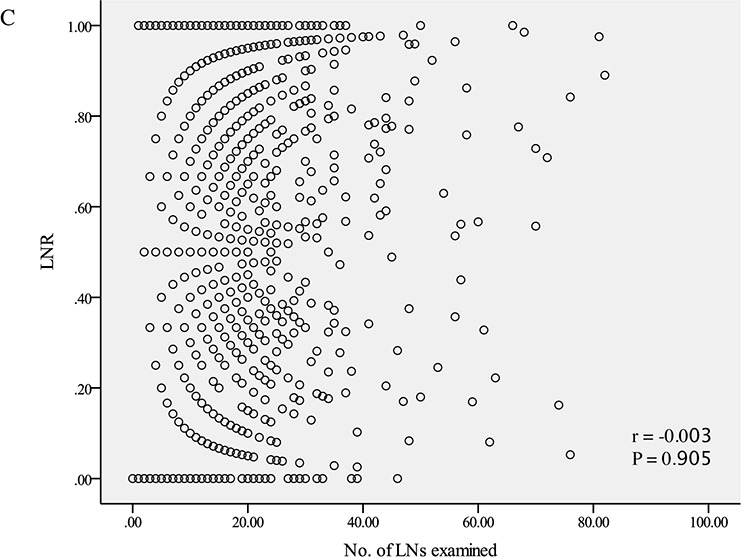
The correlation between number of LNs examined, positive lymph node count and lymph node ratio The scatter plots demonstrate the correlation between the number of lymph nodes (LNs) examined, positive lymph node (PLN) count, and lymph node ratio (LNR). **A.** Positive correlation between PLN counts and the number of LNs examined (*r* = 0.753, *P* < 0.001); **B.** Positive correlation between the LNR and PLN counts (*r* = 0.540, *P* < 0.001). **C.** No significant correlation exists between LNR and the number of LNs examined (*r* = −0.003, *P* = 0.905).

Pearson's correlation analysis showed that the PLN count significantly correlated with the number of dissected LNs (*r* = 0.753, *P* < 0.001, Figure 3A), and there was a significant positive correlation between LNR and PLN count (*r* = 0.540, *P* < 0.001, Figure 3B). However, no correlation between LNR and the LND number was observed (*r* = 0.003, *P* = 0.905, Figure 3C). Furthermore, in the proportion of patients classified as N0, we found no significant difference between subgroups divided according to number of LNs examined (“15–30” vs. “>30”) (*P* = 0.953), but the proportion of patients with N1, N2, and N3 stages increased significantly as the number of total LNs examined rose (*P* < 0.001).

### N stage migration

Table 3 shows the analysis of the N stage migration effect. In M0 GC patients with the same number of positive nodes, prognosis may differ dramatically when the number of nodes examined is insufficient (<15); this is referred to as the “stage migration effect” or “inappropriate understaging” [18, 19]. However, in DMGC patients, changes in the number of positive regional LNs merely alter N stage diagnosis but not overall staging according to the current AJCC pathologic tumor-node-metastasis (pTNM) system [20, 21]. Therefore, it is necessary to determine whether the migration effect still applies to DMGC.

**Table 3:** N stage migration analysis Survival impact of undergoing lymph node dissection and the number of dissections in N0, N1, and N2 stage in distant metastatic gastric cancer patients.

A. Univariate analysis										
UVA Factor	N0			N1			N2		
	MS (m)	3-YSR (%)	*P*	MS (m)	3-YSR (%)	*P*	MS (m)	3-YSR (%)	*P*
LND			0.000			0.001	NA		
Not performed	9.0	12.9	Ref.	7.0	4.2	Ref.	NA		
Performed	19.0	35.6	0.000	13.0	18.9	0.001	10.0	11.3	NA
LND number									
1–6	16.0	21.2	0.037	7.0	11.3	0.059	NA		
7–15	23.0	46.1	0.000	11.0	19.7	0.000	8.0	8.7	Ref.
16–30	31.0	38.4	0.001	13.0	30.5	0.000	10.0	14.3	0.015
31+	NA	65.8	0.001	17.0	57.3	0.000	21.0	3.7	0.100
Abbreviations: UVA, univariate analysis; MS, median survival; m, months; YSR, year survival rate; LND, lymph node dissection; NA, not applicable; Ref, reference.									

B. Multivariate analysis						
MVA Factor	N0		N1		N2	
	HR	95% CI	HR	95% CI	HR	95% CI
All patients						
LND (vs. Not Performed)	0.608**	0.422–0.875	0.590**	0.418–0.832	NA	
Patients with LND						
LND number^a^	0.983	0.954–1.013	0.963**	0.947–0.978	0.981**	0.966–0.995

a Continuous variable

** *P* < 0.01

Abbreviations: MVA, multivariate analysis; HR, hazard ratio; CI, confidence interval; LND, lymph node dissection; NA, not applicable.

Since the Pearson's correlation and Chi-square tests revealed that the LND number could influence the PLN count and N stage diagnosis, we performed UVA (Table 3A) and MVA (Table 3B) on N0, N1, and N2 stage patients to test the potential effect of N stage migration caused by LND as well as the number of dissections performed. For patients with N0 stage, undergoing LND correlated with better survival both in UVA and MVA. However, the number of LNs dissected was not an independent factor for favorable survival (*P* = 0.256). As we separately analyzed the risk factors for patients of N1 and N2 stages, having undergone LND and a higher number of dissections performed both correlated with increased survival in UVA and MVA.

### Survival impact of LND and number

Since metastatic LN variables could predict prognosis, and a greater LND number was associated with a more accurate diagnosis of N stage, we inquired whether undergoing LND and experiencing a greater number of dissections are detrimental to survival owing to more operative complications. Thus, we conducted survival analyses in all DMGC patients as well as node-positive patients separately.

Compared with those who did not undergo LND, the median survival and 3-YSR were significantly better in patients who underwent LND among all DMGC patients on UVA (Figure 2D, Table 4). CSS improvements were also positively associated with the number of dissected LNs. In node-positive patients (Table 4), similar CSS outcomes were observed. Cox regression (Table 5) also revealed that undergoing LND (*P* = 0.020) and a higher number of dissected LNs (*P* < 0.001) were associated with improved survival both in the entire cohort and in the LND subgroup alone.

**Table 4:** Survival impact of lymph node dissection according to univariate analysis

Factor	All patients			Patients with positive nodes		
	MS (m)	3-YSR (%)	P	MS (m)	3-YSR (%)	*P*
**LND**						
Not performed	8.0	8.1	Ref.	7.0	4.1	Ref.
Performed	11.0	16.8	0.000	11.0	14.5	0.009
**LND number**						
1–6	12.0	13.9	0.003	11.0	11.3	0.040
7–15	10.0	17.6	0.000	10.0	14.0	0.013
16–30	11.0	17.2	0.000	11.0	15.9	0.002
31+	15.0	19.4	0.000	14.0	16.6	0.000

Survival impact of undergoing lymph node dissection and the number of dissections as determined by univariate analysis in all distant metastatic gastric cancer patients, and patients with positive nodes.

Abbreviations: LND, lymph node dissection; MS, median survival; m, months; YSR, year survival rate; Ref, reference.

**Table 5:** Survival impact of lymph node dissection according to multivariate analysis

Item	All patients				Patients with positive nodes			
	All patients		With LND		All patients		With LND	
	HR	95% CI	HR	95% CI	HR	95% CI	HR	95% CI
**Age**^a^	1.005*	1.001–1.009	1.005*	1.000–1.010	NS		NS	
**T stage** (vs. T0–T2)	1.276**	1.115–1.461	NS		1.202*	1.037–1.392	NS	
**Grade** (vs. I–II)	1.261**	1.085–1.464	1.222*	1.031–1.448	1.250*	1.053–1.483	NS	
**Tumor extent** (vs. Localized)								
Further extent	1.459**	1.155–1.843	1.616**	1.223–2.134	1.589**	1.214–2.080	1.583**	1.184–2.116
**Mets at diag** (vs. DNs)								
OPI	1.326**	1.125–1.562	1.255**	1.052–1.496	1.198*	1.006–1.427	NS	
OPI and DNs	1.433**	1.132–1.813	NS		1.405**	1.088–1.815	NS	
**Radi & Surgery** (vs. Surgery alone)	0.724**	0.615–0.853	0.709**	0.593–0.848	0.637**	0.530–0.766	0.644**	0.531–0.781
**LND** (vs. Not performed	0.811*	0.680–0.967	NA		0.628**	0.456–0.864	NA	
**LND**^a^	NA		0.969**	0.959–0.978	NA		0.969**	0.960–0.979
**PLN**^a^	NA		1.041**	1.029–1.052	NA		1.037**	1.025–1.050

Survival impact of undergoing lymph node dissection and the number of dissections as determined by multivariate analysis in all distant metastatic gastric cancer patients, and patients with positive nodes.

a Continuous variable

* *P* < 0.05

** *P* < 0.01.

Abbreviations: HR, hazard ratio; CI, confidence interval; LND, lymph node dissection; PLN, positive lymph node; NA, not applicable; NS, not significance; Mets, metastasis status; diag, diagnosis; OPI, organs or peritoneal involved; DNs, distant nodes; Radi, radiation; Surg, surgery.

For node-positive patients, both LND (*P* = 0.004) and number of dissected nodes (*P* < 0.001) were significantly associated with postoperative survival benefits as well.

## DISCUSSION

This is the first study to systematically evaluate the clinical implications of accurately identifying LN status in DMGC patients based on data from a large public database. Our data showed that obtaining LN metastasis status is crucial for DMGC patients. On one hand, patients with N0 and N1 pathological diagnoses have a significant survival advantage. On the other hand, inclusion of radiation therapy, which was thought to have a significant survival benefit on locally advanced gastrointestinal cancer when combined with chemotherapy, [22, 23] had no significant impact on the survival in DMGC patients with N0 stage, and its therapeutic benefit was only apparent in patients with pathologically confirmed positive metastatic LNs based on our data (Supplementary Table S3). Therefore, accurate identification of positive nodes in patients with DMGC should be considered essential for treatment guidance.

The number of dissected LNs was considered a main factor contributing to staging accuracy in M0 patients. However, in DMGC patients, we also found that examining fewer LNs could result in understaging of the N category. Furthermore, the Chi-square test revealed that a minimum of 15 LNs should be examined for an accurate identification of positive metastatic nodes, and a higher number of LNs examined was linked with more accurate N stage classification. This finding is consistent with AJCC recommendation for curative GC, which states that >15 LNs should be evaluated for correct classification under the current TNM staging system. [20]

To further explore N stage migration as a confounding factor for survival analysis, MVA was performed on N0, N1, and N2 stage subgroups. Our results suggested that LND administration and number were both important confounders of survival analysis, especially in patients with the same positive nodal stages; this may have been caused by N stage misclassification.

Extended lymphadenectomy is regarded to have a therapeutic benefit for regional disease control in M0 patients. [19, 24] However, resection of more LNs may cause expanded tissue damage. Therefore, determining of whether undergoing LND and greater LND numbers can increase patients' mortality rates would help expose the utility of LND in clinical practice. In order to explore the potential survival impact of performing LND in DMGC patients, we analyzed the impact of undergoing LND as well as the number of dissections on CSS in the entire dataset, as well as in patients with positive metastatic node. We observed a better CSS associated with LND administration and a greater number of dissected LNs. LND administration and number were both independent prognostic factors in DMGC patients. This further illustrated the necessity and prudence of using LND information in DMGC patients who underwent palliative surgery.

Several reports [10, 13, 25–27] revealed that, compared to N stage and absolute PLN number, LNR is a more accurate prognostic indicator in M0 GC patients. In this study, we also demonstrated that LNR was superior to PLN and N stage, not only because LNR was an independent prognostic predictor while PLN was not, but also because LNR better discriminated patients' prognostic risk profiles in those of the same N stage. Additionally, we found that the LNR value could be used as a potent predictor of metastatic status of patients' overall LNs regardless of the total number of LNs examined, based on Pearson correlation test results. These data indicated that LNR is a more practical and suitable clinical prognostic indicator in DMGC patients than N stage.

There is no consensus on an optimal cut-off value for LNR. In the present study, the mean LNR was 0.59; hence, we assigned 0.6 as the cut-off value. However, we also evaluated LNR at incremental cut-off points (0.2, 0.4, 0.6, and 0.8), and found that survival decreased significantly as the cut-off point increased. Therefore, whether these LNR classification methods are appropriate in patients with DMGC requires further evaluation in a larger, prospective, randomized clinical trial.

The limitations of our study, aside from potential selection bias because of its retrospective nature, were mostly associated with the use of the SEER registry. First, the exact LND number depended on accurate identification of LNs in the resected specimen. However, because N stage does not correlate with overall staging for M1 patients, it is unknown whether diagnosing pathologists would have identified as many LNs as possible, as would be the case for M0 patients. Second, several aspects of pathology-specific covariates critical for survival evaluation, such as perineural invasion and vascular invasion, were missing from the SEER database. The effect that these variables have on outcomes may obfuscate that of LN parameters evaluated in the present study. Third, palliative chemotherapy is one of the most important prognostic factors. Since systemic chemotherapy for DMGC patients is mandatory, we assumed that all patients underwent systemic chemotherapy, although this information was not included in the SEER registry. Fourth, the heterogeneity of the SEER population would also call for cautious interpretation. Healthier patients with better prognoses were more likely to receive locoregional treatments. Moreover, the outcome of lymphadenectomy depends on the experience of the surgeons across different institutions. Accordingly, data obtained by surgeons at high-volume institutions could be overrepresented in the dataset. Despite these limitations, we are still confident that our findings elucidated the clinical significance of accurate identification of LN status and LNR in DMGC.

In conclusion, proper determination of LN metastatic status by LND is of high clinical significance. At least 15 LNs should be evaluated for precise identification for node-positive patients, and as many LNs as possible should be removed and examined to avoid N stage migration and improved regional disease control. Since there are no randomized data to validate this finding to date, and because other relevant patient-stratifying data are missing from the SEER database, caution must be exercised before applying LND for the management of DMGC patients. However, our findings provide an important basis to initiate well-controlled prospective clinical trials that could address the role of LND in DMGC patients in a more definitive fashion. Moreover, LNR was superior to PLN count as a prognostic predictor. Classification according to LNR can avoid N stage migration related to the AJCC staging system. Clinically, using LNR can better stratify survival of surgically treated patients with DMGC, reducing the number of LNs needed for accurate staging.

## MATERIALS AND METHODS

### Data collection and patient inclusion criteria

Data were obtained from the publicly available version of the SEER database released in April 2014, which consisted of 18 population-based cancer registries covering approximately 27.8% of the population of the United States. The National Cancer Institute's SEER*Stat software (Surveillance Research Program, National Cancer Institute SEER*Stat software, www.seer.cancer.gov/seerstat; Version 8.1.5) was used to access the database. Detailed patient inclusion and exclusion criteria are shown in Figure 1.

### Demographic and clinicopathological variables

Patients' demographic and clinicopathological variables, including 13 factors, were retrieved from the SEER database. The LNR was calculated by dividing the number of positive LNs by the total number of LNs dissected when at least 1 LN was examined. Among these factors, sex, race, tumor grade, histologic type, T or N stage, primary site, tumor extension, tumor metastatic status, and treatment type were considered categorical variables. Continuous variables including age, LND number, and LN variables (PLN and LNR) were binned or categorized. The subgroups created from the binning of these variables are shown in Supplementary Table S4.

### Follow-up and survival endpoints

The primary endpoint in this study was gastric CSS, defined as the period from diagnosis to death due to GC. Data of patients who died from other causes or who were alive on the date of their last follow-up were censored.

### Statistical analyses

A comparison of the categorical variables between LND subgroups was conducted using Pearson's χ^2^ test. Continuous variables were compared using the Students *t*-test. Pearson's correlation coefficient was used to study the relationships between PLN count, LNs examined number, and LNR. The Kaplan-Meier method [28] was used to calculate the actual survival rate and to plot survival curves, followed by the log-rank test [29] for UVA. MVAs were performed using the Cox regression model with stepwise regression. [30] Of note, LND number, PLN count, LNR, and patient age, which were analyzed as categorical variables on UVA, were considered continuous variables in the multivariate model. Categorical factors found to be significant (*P* < 0.05) in the UVA (Supplementary Table S3, S5–S6) combined with the above-mentioned continuous variables were analyzed using MVA. The LND number and PLN count were combined to determine the survival impact of undergoing LND as well as the number of dissections performed, whereas PLN and LNR were included simultaneously to determine which were independent survival predictors. Hazard ratios (HRs) and 95% confidence intervals were calculated, with an HR of <1.0 indicating survival benefit. N stage was not included in MVA because it was simply a manifestation of incremental PLN counts. All statistical analyses were performed using SPSS ver.19.0 (SPSS Inc., Chicago, IL), and *a* value of *P* < 0.05 indicated statistical significance.

## SUPPLEMENTARY TABLES


