# Supplementary material for: Clinical significance of accurate identification of lymph node status in distant metastatic gastric cancer
Source: Oncotarget. 2015 Oct 19;7(1):1029–41. doi: 10.18632/oncotarget.6009 (PMC4808049; doi:10.18632/oncotarget.6009)
Supplement: Supplementary file 1 [file oncotarget-07-1029-s001.pdf]

SUPPLEMENTARY TABLES

Supplementary Table S1: Patients' basic characteristics

		Number	Percent%
**Age**	18–65	954	50.5
	66+	935	49.5
**Sex**	Male	1131	59.9
	Female	758	40.1
**Race**	White	1301	68.9
	American Indian/Alaska Native	15	0.8
	Asian or Pacific Islander	320	16.9
	Black	248	13.1
	Unknown	5	0.3
**Grade**	Well/Moderately	329	17.4
	Poorly/Undifferentiation	1428	75.6
	Unknown	132	7.0
**Histology**	Carcinoma (NOS)	33	1.7
	Carcinoma (Diffuse/Linitis plastica)	142	7.5
	Signet ring carcinoma	534	28.3
	AC (Intestinal/Mucinous/ Mixed cell/ NOS)	1180	62.5
**Tumor site**	Fundus	54	2.9
	Body	182	9.6
	Antrum	486	25.7
	Cardia	299	15.8
	Pylorus	68	3.6
	Curve (Greater/Less)	297	15.7
	Other (Overlapping site/ NOS)	503	26.6
**T stage**	Less (T0–T2)	696	36.8
	Deeper (T3–T4)	1120	59.3
	TX	73	3.9
**N stage**	Negative (N0)	290	15.4
	Positive (N1–N3)	1497	79.2
	NX	102	5.4
**No. of LNs dissected**	0	272	14.4
	1–6	312	16.5
	7–15	571	30.2

(*Continued*)

		Number	Percent%
	16–30	495	26.2
	31+	135	7.1
	No specific value	104	5.5
**PLN**	0	151	8.0
	1–6	589	31.2
	7–15	491	26.0
	16+	292	15.5
	No specific value	366	19.4
**LNR**	≤0.2 [0–0.2]	302	16.0
	≤0.4 (0.2–0.4]	195	10.3
	≤0.6 (0.4–0.6]	215	11.4
	≤0.8 (0.6–0.8]	246	13.0
	≤1.0 (0.8–1.0]	553	29.3
	Not available	378	20.0
**Tumor extent**	Further extension	131	6.9
	Regional	389	20.6
	Localized	1296	68.6
	No value	73	3.9
**Metastatic status at diagnosis**	"Organs or peritoneal involved" + "distant nodes"	177	15.6
	Organs or peritoneal involved	1413	74.8
	Distant nodes	294	9.4
	No value	5	0.3
**Treatment**	Surgery only	1560	82.6
	Combined surgery and radiation	302	16.0
	No value	27	1.4
**Total**			1889

Supplementry Table S1 shows the information of DMGC patients basic characteristics included in our study. Abbreviations: NOS, not otherwise specified; AC, adenocarcinoma; PLN positive lymph node; LNR, lymph node ratio.

Supplementary Table S2: Patient characteristics according to lymph node dissection

Item		*P*	LND			
			Not Performed *N* = 272		Performed *N* = 1593	
			No.	Per (%)	No.	Per (%)
**Age**	>65	0.743	138	50.7	788	49.5
**Gender**	Male	0.255	154	56.6	963	58.8
**Race**	White	0.098	197	72.4	1087	68.2
	Black		40	14.7	206	12.9
	Asian		32	11.8	285	17.9
	Indian		2	0.7	11	0.7
**Grade**	III-IV	0.853	178	65.4	1242	78.0
**Histology**	Carcinoma (NOS)	0.000	12	4.4	18	1.1
	Carcinoma (DT/LP)		14	4.4	126	7.9
	SRC		80	29.4	448	28.1
	Adenocarcinoma		166	61.0	1001	62.8
**Tumor site**	Fundus	0.157	8	2.9	45	2.8
	Body		25	9.2	156	9.8
	Antrum		67	24.6	416	26.1
	Curve		33	12.1	263	16.5
	Cardia		40	14.7	256	16.1
	Pylorus		9	3.3	59	3.7
	Other		90	33.1	398	25.0
**T stage**	Deeper (T3–T4)	0.107	128	47.1	986	61.9
**N stage**	Positive (N1–N3)	0.000	56	20.6	1457	91.5
**Tumor extent**	Further	0.000	37	13.6	90	5.6
	Regional		53	19.5	334	21.0
	Localized		135	49.6	1154	72.4
**Mets at diag**	OPI & DNs	0.000	40	14.7	134	8.4
	OPI		211	77.6	1183	74.3
	DNs		21	7.7	272	17.1

Supplementry Table S2 represents the difference of clinicopathological variables between those who underwent LND and those who did not.
Abbreviations: LND, lymph node dissection; Per, percent; NOS, not otherwise specified; DT, diffuse type; LP, linitis plastica; SRC, signet ring carcinoma; Mets, metastasis status; diag, diagnosis; OPI, organs or peritoneal involved; DNs, distant nodes.

Supplementary Table S3: Univariate survival analyses of categorical variables in nodal metastasis status subgroups

Factor	All patients		Patients with LND	
	Negative (N0)	Positive (N1–N3)	Negative (N0)	Positive (N1–N3)
Sex (vs. Male)	0.074	0.089	0.052	0.049
Race (vs. White)	0.233	0.404	0.408	0.404
T stage (vs. T0–T2)	0.000	0.000	0.003	0.000
Grade (vs. I–II)	0.002	0.002	0.013	0.006
Histology (vs. AC)	0.140	0.029	0.008	0.049
Tumor site (vs. Body)	0.002	0.000	0.030	0.000
Tumor extension (vs. Localized)	0.000	0.000	0.000	0.000
Metastasis status (vs. Distant nodes)	0.000	0.000	0.003	0.000
Radi & Surg (vs. Surgery only)	0.363	0.000	0.874	0.000

Supplementry Table S3 shows *P* values of clinicopathological variables tested by univariate survival analysis in the subgroups of different nodal metastasis status.
Abbreviations: LND, lymph node dissection; AC, adenocarcinoma; Radi, radiation; Surg, surgery.

Supplementary Table S4: Demographical and clinicopathologic variables definition

Variable	Bins created
**Age**	18–65, >65
**Sex**	Men, Women
**Race**	nH-white, nH-blacks, nH-Asians/Pacific Islanders, American Indian/Alaska native
**Tumor grade**	Well/moderately differentiated, Poorly/undifferentiated, unknown
**Histology type**	Unspecified carcinoma, DT carcinoma, SR carcinoma, Adenocarcinoma, Unknown
**Tumor site**	Cardia, Fundus, Body, Antrum, Curve, Pylorus, Other (overlapping or unspecified)
**T stage**	T0–T2, T3–T4, TX
**LNs metastasis status**	Negative (N0), Positive (N1–N3)
**N stage**	N0, N1, N2, N3, NX
**Tumor extentison**	Localized (limited in gastric and serosa or gastric ligaments or the omentum invasion), Regional (other alimentary system organs involved), Further extension
**Tumor mets at diag**	Merely DNs metastasis, Merely OPI, DNs plus OPI
**Treatment**	Surgery onlySurgery combined with radiation (Radi & Surg)
**No. of LNs examined**	0, <7, 7–15, 16–30, >30
**PLN counts**	0,< 7, 7–15, >16
**LNR[b]**	0–0.6, >0.6
**LNR[m]**	[0–0.2], (0.2–0.4], (0.4–0.6], (0.6–0.8], (0.8–1.0]

Supplemententry Table S4 shows the classification of variables retrieved from SEER database.
[b]Binary variable;
[m]Multi-category variable.
Abbreviations: nH, non-Hispanic; DT, diffuse type; SR, signet ring; Mets, metastasis status; diag, diagnosis; DNs, distant nodes; OPI, organs or peritoneal involved; PLN, positive lymph node; LNR, lymph node ratio; Radi, radiation; Surg, surgery.

Supplementary Table S5: Univariate survival analyses of categorical variables in all patients and patients with lymph node dissection

Factor	All patients	Patients with LND
**Sex** (vs. Male)	0.014	0.008
**Age** (vs. 18–65)	0.012	0.065
**Race** (vs. White)	0.846	0.640
Black	0.912	0.496
Asian	0.671	0.595
Indian	0.445	0.328
**T stage** (vs. T0–T2)	0.000	0.000
**Grade** (vs. I–II)	0.000	0.000
**Histology** (vs. AC)	0.179	0.002
Carcinoma (NOS)	0.600	0.525
Carcinoma (DT/LP)	0.140	0.107
SRC	0.001	0.000
**Tumor Site** (vs. Body)	0.004	0.000
Fundus	0.513	0.606
Antrum	0.644	0.908
Curve	0.364	0.248
Cardia	0.005	0.030
Pylorus	0.978	0.540
Other	0.015	0.014
**Tumor extent** (vs. Localized)	0.000	0.000
Regional	0.000	0.001
Further extent	0.000	0.000
**Mets at diagosis** (vs. DNs)	0.000	0.000
OPI	0.000	0.000
OPI and DNs	0.000	0.000
**Radi & Surg** (vs. Surgery alone)	0.000	0.000

Supplementry Table S5 shows P values of clinicopathological variables tested by univariate survival analysis.
Abbreviations: LND, lymph node dissection; AC, adenocarcinoma; NOS, not otherwise specified; DT, diffuse type; LP, linitis plastica; SRC, signet ring carcinoma; Mets, metastasis status; OPI, organs or peritoneal involved; DNs, distant nodes; Radi, radiation; Surg, surgery.

Supplementary Table S6: Univariate survival analyses of categorical variables in subgroups of different N stages

Factor	All patients			Patients with LND		
	N1	N2	N3	N1	N2	N3
Sex (vs. Male)	0.302	0.473	0.397	0.163	0.472	0.397
Race (vs. White)	0.485	0.741	0.619	0.464	0.719	0.619
T stage (vs. T0–T2)	0.000	0.015	0.964	0.000	0.018	0.964
Grade (vs. I–II)	0.025	0.246	0.116	0.086	0.224	0.116
Histology (vs. AC)	0.124	0.000	0.186	0.203	0.000	0.186
Tumor site (vs. Body)	0.026	0.007	0.785	0.021	0.007	0.785
Tumor extension (vs. Localized)	0.002	0.004	0.026	0.005	0.004	0.026
Metastasis status (vs. Distant nodes)	0.000	0.041	0.436	0.001	0.049	0.436
Radi & Surg (vs. Surgery only)	0.000	0.001	0.123	0.000	0.001	0.123

Supplementry Table S6 shows *P* values of clinicopathological variables tested by univariate survival analysis in the subgroups of different N stages.

Abbreviations: LND, lymph node dissection; AC, adenocarcinoma; Radi, radiation; Surg, surgery.
